# Annotations

**Published:** 1891-10-31

**Authors:** 


### Annotations.

**Brain Rust.**

Sir James Crighton Browne has been lecturing to the public on "Brain Rust." He evidently believes, as do so many of his fellow-countrymen, that it is better to wear out than to rust out. And so do we. But we also believe that it is better to wear out than to be prematurely disintegrated. The quarrel nowadays is not between those who wear out their brains by steady and solid work, and those who rust them out by idleness, but between those who wear them out soberly and steadily on the one hand, and those who prematurely reduce them to pulpy imbecility by excessive labour on the other. We agree with Sir James Crighton Browne that brain work and plenty of it is eminently conducive to vigorous health and long life. Brain work, indeed, is quite as essential as muscular activity. Such work not only makes a man clever and bright and successful, but it increases the appetite, improves the digestion, promotes the circulation of both blood and lymph, and generally exalts the whole of the physiological activities of the body. But it seems to us that Sir James Crighton Browne has not marked out with lines of sufficient distinctness the other aspects of the question. Brain work is often excessive in these days, and brain waste, therefore, much too rapid. The results are experienced in three obvious and injurious particulars, namely, in dyspepsia, irritability of temper, and sleeplessness. Any man who begins to find himself suffering from these three symptoms, should moderate his brain work immediately, and seek competent medical advice. The most dangerous excesses which any man can indulge in are those which tell upon the brain and the nervous system. The nervous system, as a recent writer has well said, is the very centre and essence of man. The trunk and limbs, the bloodvessels and the digestive organs, the muscles and the bones are mere accessories, adjuncts, and environment. The essence of the business for man is the brain, with its continuation in the spinal cord and the peripheral nervous system. Clearly, therefore, the brain must work vigorously and well, seeing that it and it alone is the real and actual man. But as clearly it must not work in such a way as to seriously and permanently injure itself, for that means the destruction and death of that which is the actual man. What we want to arrive at is a just balance between mental and physical activity, an active and healthy brain in an active and healthy body, a vigorous thinking power utilising an energetic and wholesome environment, and a healthy environment ministering to the vigorous thinking power.

**Dangerous Practitioners.**

An infant died last week at Birmingham over whose body it was found necessary to hold a coroner's inquest. According to the evidence, the baby died of convulsions; and the cause of the convulsions seems to have been the pain and swelling of the gums resulting from teething. A medical man who gave evidence said that if the gums had been properly lanced the pain would have been relieved and the infant's life probably saved. The person who attended the child, and who was believed by the parents, at the time, to be a doctor, said he had not lanced the baby's gums, but he had ordered it a pinch of snuff, presumably with the homœopathic intention of curing one set of convulsions by inducing another set. How came this man, who was not a doctor, to be called in to a dangerous case of convulsions, exactly as if he had been a doctor? The brass plate did the business. The man seems to have had a brass plate on his door with the name of a fully qualified medical practitioner inscribed thereon. The parents of the convulsed child called in the occupier of the house in the full belief that it was his name which was inscribed upon the brass plate. The real owner of the name admitted that in addition to carrying on practice at his own house, he had two branch medical establishments which were conducted in his name by men who had no qualifications. All these three men deserve very hard names, and very severe punishment. They deceive the public; and their deception sometimes, perhaps often, results in loss of life. Are they not responsible, every one of them, for such loss of life? And if they are, is it manslaughter, or the more shocking crime of deliberate murder, with which they should be charged? With the means now at the disposal of the public, it is difficult to punish such men; and it is difficult, because the ordinary persons who set the law in motion that is fully qualified neighbouring practitioners, usually have something to gain by the punishment of the unqualified and their abettors. But it should not be left to interested parties to set the law in motion in such cases. The public have the right to be protected against unqualified medical practice; and there is a very powerful organisation, whose duty it is to protect it. That organisation is the General Medical Council. The Council is a body in whom the Government, the public, and the medical profession alike have confidence. It has done a certain amount of work with regard to medical education and registration very well indeed; and it has held the balance very evenly between public and medical interests. But it ought now to go a few steps further than it has hitherto gone. Illicit medical practice is a public danger; a danger which results in a great many deaths during the year. It is, moreover, a grievous scandal to civilization. All that is wanted is that the Medical Council shall boldly put down its foot. The Government and the press will pay heed to it when they will not pay heed to associations of interested medical practitioners. Such men as the Birmingham doctor, who has allowed unqualified persons to practice in his name, should be first admonished; and if they still persist in their wicked practices, they should be struck off the Medical Register. The unqualified ones, when caught red-handed in their dangerous work, should be punished for manslaughter or murder.

**Sleeplessness and Suicide.**

If anything can excuse suicide it is sleeplessness. A jury of College masters and Eton residents the other day decided that Dr. Philip Herbert Carpenter, Science Master at Eton, had killed himself by the administration of chloroform while suffering from temporary insanity. It was given in evidence that Dr. Carpenter had been a martyr to sleeplessness. The deceased gentleman had had a severe attack of influenza, and this had been followed by great restlessness and want of sleep; both of which conditions indicated a distressing exhaustion of the nervous system. Dr. Carpenter visited the Isle of Wight, and improved decidedly in health, but he appears to have returned to his work too soon, for he had scarcely well begun when sleep again deserted him. On walking to the school last Wednesday he said he felt his brain reeling, and that he had had no sleep. Indigestion, probably partly the cause and partly the effect of the insomnia, added its torments to Dr. Carpenter's mind. He grew desperate, and in a moment of frenzied despair destroyed himself. He was but thirty-nine years of age, and has left a widow and four young children. This is the kind of tragedy that modern times produce. It all comes, ultimately, from too much work of the brain and too little work of the muscular system. Have doctors, schoolmasters, and university professors ever considered the fact that horses and cattle, dogs, and other animals practically never suffer from insomnia? Of course, when attacked by acute disease, or experiencing sharp pains, they are wakeful; but at ordinary times sleeplessness is a thing unknown amongst them. The farm labourer and the navvy are like the horse and the dog—they do not, in a regular way, know what sleeplessness is. Now is it not the case that science and culture ought to diminish for us the ills of life, not to increase them? Culture and science that produce among the cultured and scientific classes a wide range of ills, like sleeplessness and dyspepsia, are not benefits but injuries. They do not mark real progress, but retrogression. If our men of science—physicians and teachers of all kinds—were men of really able minds, they would establish among us methods of culture and ways of living which would increase the health and enduring power of the brain, not destroy it. The readiness to learn is one of the surest of all marks of true intellectual capacity. Does the average University professor, school teacher, or doctor show himself in a constant state of willingness to receive instruction? Not in the least. He shows himself willing to bow to the authority of a few great names. But instruction that comes to him in homely garb, or from any other than recognised seats of authority, he flouts. It is an infinite pity. What a splendidly capable race we English-speaking peoples would be if we would always keep open minds, and always act up to our best judgment and conscience. A tragedy like that at Eton is quite an every-day occurrence among us now, and for no other reason but that we have not the intellectual strength to stand against the schoolmaster interest which crams and kills our boys and young men for no other purpose but to serve its own private ends.

